# Spatial Autocorrelation, Source Water and the Distribution of Total and Viable Microbial Abundances within a Crystalline Formation to a Depth of 800 m

**DOI:** 10.3389/fmicb.2017.01731

**Published:** 2017-09-19

**Authors:** E. D. Beaton, Marilyne Stuart, Sim Stroes-Gascoyne, Karen J. King-Sharp, Ioana Gurban, Amy Festarini, Hui Q. Chen

**Affiliations:** ^1^Canadian Nuclear Laboratories (formerly Atomic Energy of Canada Limited), Chalk River Laboratories, Deep River ON, Canada; ^2^Department of Earth and Environmental Sciences, University of Ottawa, Ottawa ON, Canada; ^3^3D Terra, Montreal QC, Canada

**Keywords:** microbial ecology, fracture water, groundwater, cell density distribution, modeling, spatial autocorrelation

## Abstract

Proposed radioactive waste repositories require long residence times within deep geological settings for which we have little knowledge of local or regional subsurface dynamics that could affect the transport of hazardous species over the period of radioactive decay. Given the role of microbial processes on element speciation and transport, knowledge and understanding of local microbial ecology within geological formations being considered as host formations can aid predictions for long term safety. In this relatively unexplored environment, sampling opportunities are few and opportunistic. We combined the data collected for geochemistry and microbial abundances from multiple sampling opportunities from within a proposed host formation and performed multivariate mixing and mass balance (M3) modeling, spatial analysis and generalized linear modeling to address whether recharge can explain how subsurface communities assemble within fracture water obtained from multiple saturated fractures accessed by boreholes drilled into the crystalline formation underlying the Chalk River Laboratories site (Deep River, ON, Canada). We found that three possible source waters, each of meteoric origin, explained 97% of the samples, these are: modern recharge, recharge from the period of the Laurentide ice sheet retreat (*ca*. ∼12000 years before present) and a putative saline source assigned as Champlain Sea (also *ca*. 12000 years before present). The distributed microbial abundances and geochemistry provide a conceptual model of two distinct regions within the subsurface associated with bicarbonate – used as a proxy for modern recharge – and manganese; these regions occur at depths relevant to a proposed repository within the formation. At the scale of sampling, the associated spatial autocorrelation means that abundances linked with geochemistry were not unambiguously discerned, although fine scale Moran’s eigenvector map (MEM) coefficients were correlated with the abundance data and suggest the action of localized processes possibly associated with the manganese and sulfate content of the fracture water.

## Introduction

A goal of ecology is to relate population densities from within a region of interest to local or regional environmental conditions, however, analyses of spatially distributed sampling locations can be complicated by autocorrelation (Dormann et al., 2007; Gilbert and Bennet, 2010) or a lack of independence between nearby sampling locations. This characteristic, if not recognized, can lead to incorrect conclusions for population and environment interrelationships. When modeling population densities within a region of interest, autocorrelation can be caused by, for example, distance relationships in biological processes such as dispersal, by assuming an incorrect relationship between abundances and environment within a model, or by not accounting for an important environmental determinant that in itself is spatially structured and thus causes spatial structuring in the response (Dormann et al., 2007). Discovery of distance-relationships associated with biological processes provides an important and interesting insight on community patterns while the assumptions made when modeling population abundances can lead to incorrect conclusions by having model residuals that are not randomly distributed, and so are themselves autocorrelated (Dormann et al., 2007).

Within the volume of proposed geologic repositories for hosting waste with inventories of long-lived radionuclides, information on the microbial abundances within an undisturbed setting at depth can help formulate conceptual models for long-term subsurface dynamics over the expected inventory decay period. A microbial community is defined as an assemblage of potentially interacting taxa that co-occur over space and time (Nemergut et al., 2013). Differences in abundances over space and time can occur through a combination of processes such as by abiotic selection and biotic competition or by speciation and drift between unconnected communities (Hubbell, 2001; Vellend, 2010). Microbial distributions in natural water systems also tend to be dispersed (Bliss and Fisher, 1953; El-Shaarawi et al., 1981; Haas and Heller, 1986; Hilbe, 2011; Harrison, 2014); occurring as clusters of cells or associated with suspended particles.

**FIGURE 1:**
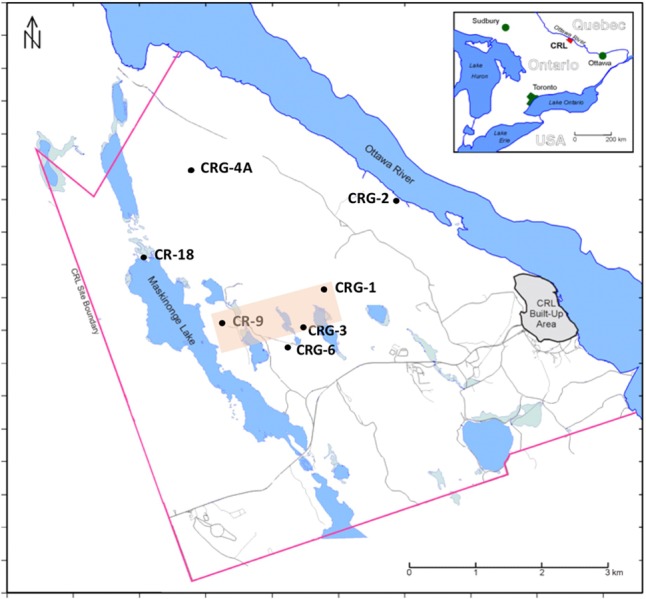
Fracture water sampling locations, Chalk River Laboratories, Deep River, ON, Canada. Boreholes CR-9 and CR-18 were drilled into the bedrock ca. 1980, boreholes CRG-1 through CRG-7 were drilled into the bedrock since 2005. All boreholes except CR-18 were sealed and isolated into multiple intervals using Westbay^TM^ Systems. Borehole CR-18 is an open borehole. The shaded area transects the boreholes used for visualizing the M3 results shown in **Figure 3**.

In this study, distributions of the total and viable count data and the geochemistry data were derived from sampling multiple saturated fractures that were accessed from boreholes drilled into overlapping bedrock assemblages underlying the Chalk River Laboratories (Deep River, ON, Canada) site. Data collection was part of a siting assessment for a potential future geologic waste management facility at the CRL site (Thompson et al., 2011). The locations of these boreholes are shown in **Figure 1**. Previous studies performed within this formation (Stroes-Gascoyne et al., 2011; Beaton et al., 2016) showed that bacterial taxa were numerically dominant in the fracture water and that these bacteria displayed nitrogen metabolism with episodes of sulfur metabolism. This finding is akin to other crystalline subsurface environments hosting microbial communities that display metabolic activity such as nitrate, iron and sulfate reduction (Kieft, 1990; Jain et al., 1997; Haveman et al., 1999; Sahl et al., 2008; Nyyssönen et al., 2012). Although the bacteria were mainly uncultured, the closest cultivated representatives were from the phenotypically diverse *Betaproteobacteria*, *Deltaproteobacteria*, *Bacteroidetes*, *Actinobacteria*, *Nitrospirae*, and *Firmicutes*. Hundreds of taxa were identified but only a few were found in abundance (>1%) across all 16S rRNA assemblages. A decay of phylogenetic similarity with distance up 1.5 km was evident within sampling locations separated by up to 5 km of rock (Beaton et al., 2016). We propose that this decay distance is related to dispersal within vertical oriented fractures. To test for the possible influence of recharge and metabolism on total and viable abundances we extend our findings for nitrogen metabolism and sulfate reduction (Stroes-Gascoyne et al., 2011) and for the distance decay of similarity (Beaton et al., 2016) by analyzing the relative influences of the fracture water on microbial abundances and viability; an aspect of this subsurface habitat that had not been evaluated previously. Isotopic analysis of the dilute fracture water indicates it is of meteoric origin – with no significant rock-water interactions (King-Sharp et al., 2016); Supplementary Figure S1 shows the stable isotope composition for hydrogen and oxygen in the fracture water relative the Vienna Standard Mean Ocean Water (VSMOW). This recharge provides a possible source of soluble species for microbial processes and is a medium for dispersal. Porewater analysis from rock cores (Peterman et al., 2016) identified nitrogen compounds within the porewater composition that were not detected within the fracture water, so despite the stable isotope compositions relative to VSMOW (Supplementary Figure S1), rock-water interactions relevant to microbial abundances may still be occurring.

To gauge interrelationships between subsurface microbial abundances with the geochemistry we combined the abundance and geochemical data from multiple sampling opportunities and performed modeling to address whether recharge can explain how subsurface communities assemble within these fractures. The chemical species within the fracture water were evaluated for their significance as explanatory variables by a multivariate approach in which the fracture water compositions were compared with the compositions of known and derived compositional end-members. The explanatory power of the end-member compositions provide insight into probable source waters and, therefore, insight into the history of recharge, mixing and other geological processes (Laaksoharju et al., 1999; Laaksoharju et al., 2008) that may have shaped the current fracture water compositions. Moran’s I was used to determine spatial autocorrelation between sampling locations and the fracture water components were evaluated by a generalized linear model (GLM) (Venables and Ripley, 2002) for their significance as possible metabolic substrates associated with microbial abundances. Positive Moran’s eigenvector map (MEM) coefficients were included as independent variables in the GLM to gauge for spatial autocorrelation within the model residuals.

## Materials and Methods

### Fracture Water Sampling and Analysis

Fracture water was collected using a Westbay^TM^ Multilevel Groundwater Monitoring System (Schlumberger Water Services). Supplementary Figure S2 shows a schematic of a borehole with an installed Westbay System^®^. This Figure illustrates how the Westbay tubing and packers isolate multiple zones within the borehole thus preventing unnatural vertical fracture water flow within the borehole itself. The tubing fluid is isolated from the formation fluid. In this arrangement, ambient formation fluid flow can pass through the annulus. From inside the tubing, formation fluid can be accessed by lowering a Westbay sampler and container assembly (also shown) to normally closed valved ports positioned between the packers. A larger schematic illustrates a deployed Westbay sampler assembly that is engaged at a selected port. Once the sampler is positioned and engaged, the remotely operated control valve in the sampler is opened to allow formation fluid from the zone to flow into the empty container. The process is monitored by observing changes in fluid pressure during the sequence of operations (see a typical trace of pressure vs. time in Supplementary Figure S2). Once the container is filled, the sampler valve is closed to seal the formation fluid inside the container at *in situ* pressure. The assembly is disengaged from the port (the port valve automatically closes) and the fluid in the sealed container is retrieved to the surface for further handling.

The fracture water sampler consists of four 250 mL stainless steel tubes connected in series by tubing and Swagelok fittings. Prior to each sampling, the tubes were sterilized by autoclave and the fittings were sterilized by washing them with 70% ethanol. Validation of the sterilization and transport procedures was performed using sterilized water and PCR with bacterial rRNA 16S primers (Muyzer et al., 1993). Since the tube assemblies contacted only the interior of the casing surface, the probability of introducing surface microbes into the sampled volumes was minimal.

The borehole locations within the study site region of interest, and their names, are shown in **Figure 1**. These sampling locations are situated between the geological boundaries created by the Maskinonge Lake fault, the Mattawa fault (Ottawa River) and by East-West trending diabase dykes that traverse the study site along the boreholes CR-9, CRG-3 and CRG-6. Fracture water was collected from sealed borehole CRG-1, CRG-2, CRG-3, CRG-4A, CRG-6 and CR-9. Fracture water from an open unsealed borehole, CR-18, was also sampled. Depths of the sampled fracture water ranged from 35 to 780 m (137 to -800 m elevation, relative to sea level).

The fracture water pH [Beckman PHI 265 pH/Temp/mV meter (Beckman Coulter, Inc.)] and conductivity [YSI Model 30 Conductivity Meter (YSI Inc., Yellow Springs, OH, United States) were measured and aliquots for elemental analysis were filtered through a 0.45 μm filter (isopore polycarbonate, Millipore, Billerica, MA, United States) then immediately preserved in nitric acid (ultra-trace grade, Seastar^TM^, Baseline^®^, Fisher Scientific, Ottawa, ON, Canada). Elemental composition of the fracture water was determined by inductively coupled plasma-mass spectrometry (ICP-MS, using either a Varian 820-MS (Agilent Technologies, Inc.) or an Element XR (Thermo Scientific)) and by inductively coupled plasma atomic emission spectroscopy (ICP-AES, Optima 3300, Perkin Elmer). Anion concentrations were determined using a Dionex 3000 ICS ion chromatograph (Dionex, Sunnyvale, CA, United States). Dissolved organic (DOC) and inorganic carbon (DIC) were determined using a Dohrmann, model Phoenix 8000-UV Persulfate TOC Analyzer (Teledyne Teckmar, Mason, OH, United States).

Total and viable microbial densities were determined by fluorescence microscopy with a Nikon E600 microscope and a Zeiss Axiophot microscope after filtering the separate stained samples onto black polycarbonate filters (Fisher Scientific, 25 mm, 0.22 μm pore size); at least fifteen fields of view and at least 300 cells were counted per filter for a coefficient of variation of 5.8% per filter. Direct counts for total cell densities were determined in triplicate 1 mL volumes – within 4 h of sampling at the formation pressure and within 1 h of opening the sample tubes including a 30 min incubation time. Total cell densities were determined using the DNA intercalating dye, Sybr Green II (Life Technologies); because separate aliquots were shipped to a another laboratory, total cell counts were also determined within 24 h of opening the sample tubes, in this case, using Acridine Orange (Sigma–Aldrich) to emulate the procedure employed at the receiving laboratory; the two dyes and two time points gave similar results. Direct counts for viable cell densities were determined in triplicate 1 mL volumes, also within 1 h of opening the sampling tubes, using dyes that are sensitive to different characteristics of viable microbial cells: the soluble 5-cyano-2,3-ditolyl tetrazolium chloride (CTC, Sigma–Aldrich) was used to evaluate respiratory activity within the microbial population as detected by the reduction of CTC to the insoluble fluorescent CTC-formazan (Schaule et al., 1993); the lipophilic cation, rhodamine-123 (R123 Sigma–Aldrich) (McFeters et al., 1998; Fuller et al., 2000) was used to evaluate cells within the microbial population that display a membrane potential difference; and carboxyfluorescein diacetate (CFDA, Sigma–Aldrich) was used to evaluate enzymatic activity (Schaule et al., 1993).

### Multivariate Mixing and Mass Balance Analysis

The fracture water components sodium (Na^+^), calcium (Ca^2+^), magnesium (Mg^2+^), bicarbonate (HCO_3_^-^), chloride (Cl^-^), sulfate (SO_4_^2-^) and the isotopes tritium, deuterium and stable oxygen, δ^18^O, were used as input data for the multivariate, mixing and mass balance analysis model (M3, performed by 3D Terra, Montreal, Quebec). The M3 model consists of four steps: a principal component analysis (PCA); selection of reference waters (end-members) followed by calculations of mixing proportions; and finally mass balance calculations (Laaksoharju et al., 1999; Laaksoharju et al., 2008).

Three end-members were found to describe the fracture water; these are referred to as: (1) ‘recharge,’ (2) ‘Champlain Sea’ (or ‘saline’), and (3) ‘glacial melt’ (not shown). The stable isotope values for the melt water end member were taken from the literature: the δ^18^O value from Frape and Fritz (1987) and Remenda et al. (1994). The deuterium value was determined by Rozanski et al. (1993). The tritium value, which governs the proportion of recharge, was considered decayed to zero. The end-member referred to as ‘Champlain Sea’ was obtained from nearby sediment pore water from this period in the site history that had a salinity of 6.1% (Torrance, 1988). The end-member referred to as modern recharge was calculated as an average of the chemistry of the upper section of boreholes CRG-2, CRG-3, CRG-4A and CRG-6-1 and CR-9-1. The software, Surfer (Golden Software), was used to create 2D cross section maps.

### Generalized Linear Model with a Negative Binomial Distribution

The replicate values for microbial cell densities and geochemistry were averaged for each borehole interval sampling location. Supplementary Figure S3 shows a comparison of quantile-by-quantile plots for the total cell count distribution against theoretical normal and negative binomial distributions. The environmental and spatial data were evaluated as explanatory variables using the glm.nb() function from the R package ‘MASS’ (Venables and Ripley, 2002). Significant variables were determined by stepwise modeling. Model selection was based on minimizing the Akaike information criterion (AIC). Analysis of variance was applied to the reduced model to determine the significance of the retained variables. Only those values with *p* < 0.05 were considered significant. All of the model results are provided in an Excel file in the Supplemental Information.

### Spatial Autocorrelation and Moran’s Eigenvector Map Coefficients

Moran’s eigenvector map were created by principle coordinates of neighbor matrices (Borcard and Legendre, 2002; Dray et al., 2006) from within the R packages ‘sdep’ and ‘adespatial’. A matrix of spatial eigenvectors was built from a distance matrix of Easting and Northing, zone 18, Universal Transverse Mercator coordinates for each borehole interval. The functions used to create the spatial weightings matrix were nbtri(), that converts the spatial coordinates of the sampling locations into a distance neighbors map, and the function nb2listw() that creates the weightings matrix from the neighbors map. The eigenvectors for positive values for Moran’s I reveal different spatial structures over the entire range of scales encompassed by the geographical sampling area. The first MEM values generated in the analyses represent broader spatial structures, and the last MEM values represent finer spatial structures. Values for Moran’s I at each sampling location were compared to a null distribution of the global Moran’s I using the function localmoran(). The resulting *z*-values were plotted to display locations with spatial correlations that were more than two standard deviations from the null mean.

## Results

### Multivariate Mixing and Mass Balance (M3) Modeling

**FIGURE 2:**
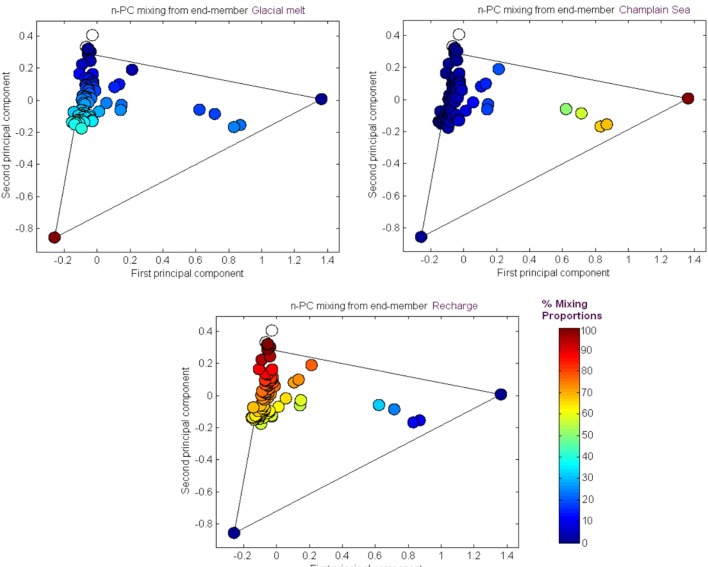
M3 Principal Component analysis of the major fracture water ions and stable oxygen δ^18^O. The results are shown in relation to the M3 modeling results for source water mixing proportions: glacial melt, saline and recharge waters.

**FIGURE 3:**
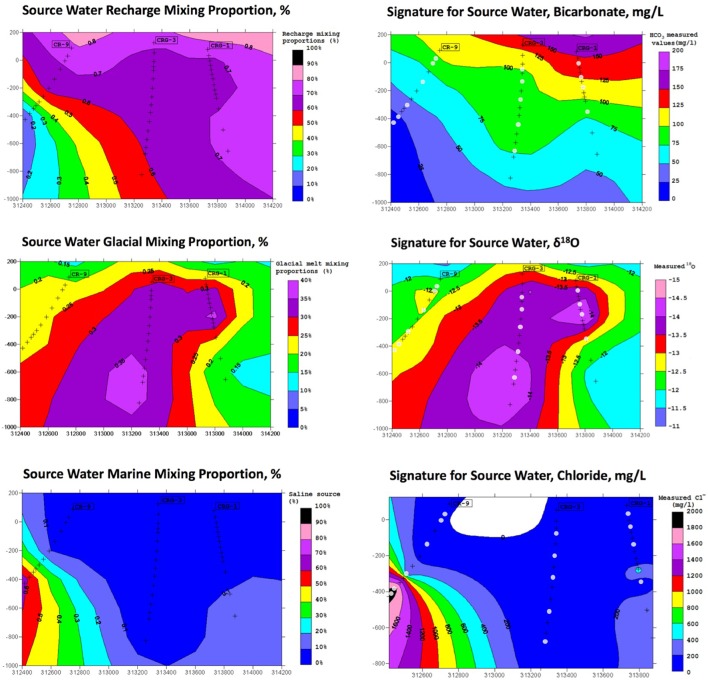
M3 results for source water end members **(left)** and a candidate signature associated with the source water **(right)**. The results are shown in cross section referenced to the boreholes CR-9, CRG-1, and CRG-3.

The results from the PCA are shown in **Figure 2**. The PCA results are displayed three times to illustrate modeling results for mixing of the three fracture water compositional end-members; these were the percent mixing proportion for glacial melt water (**Figure 2**, upper left panel), the percent mixing proportion for Champlain Sea (**Figure 2**, upper right panel), and the percent mixing proportion for modern recharge (**Figure 2**, bottom panel). The first and second principal components accounted for 71% of the variance in the geochemistry. The area encompassed by the three end-members (**Figure 2**, triangle joining the three compositional end-members) explains over 97% of the fracture water samples; most of the individual fracture water compositions plot between the three reference waters. The fracture water samples that plot outside the region of the three end members are listed as open circles (**Figure 2**, all three panels). The explanatory power of glacial melt, Champlain Sea and modern recharge may indicate that these waters have affected the present fracture water composition and thus represent historical events that could have influenced the fracture water microbial populations. The modeled mixing proportions of the three source waters suggests that fracture water sampled from boreholes CRG-1, CRG-2, CRG-3, CRG-4A, CRG-6, and CR-18 contain mainly modern recharge with a small glacial melt mixing proportion of up to ∼40%. Fracture water sampled from borehole CR-9 includes proportions from these source waters and an additional mixing proportion from a saline water source, referred to here as Champlain Sea. Fracture water accessed from intervals 11 and 12 from borehole CR-9 have mainly a saline water type signature of ∼70%. By this model, the fracture water from CR-9-3, CR-9-8 and CR-18 is a mixture of Champlain Sea, melt water and modern water. Distributions of the three possible water sources is represented in cross section in **Figure 3** (left panel) for modern recharge (top left panel), glacial melt (middle left panel) and Champlain Sea (bottom left panel). The depth and orientation profiles for boreholes CR-9, CRG-1 and CRG-3 are also shown. These boreholes form an East-West transect across the study site (depicted by the shaded area in **Figure 1**); the sampling locations within these boreholes that were used for microbial abundance determinations are shown in **Figure 3** as white dots. The visualizations were created by 2-D kriging interpolating between the sampling locations within each of these boreholes and do not account for the fractures that would provide the water flow paths throughout the rock matrix. The left-hand side of **Figure 3** shows the mixing proportion by prospective source water and the right-hand side of the **Figure 3** shows the distributions of geochemical signatures that correspond to these water sources: bicarbonate (**Figure 3**, top right panel) for modern recharge; measured δ^18^O values (**Figure 3**, middle right panel) for glacial melt water and chloride (**Figure 3**, bottom right panel) for a saline source water. These components of the fracture water, therefore, may represent a signature for source water in a GLM.

### Spatial Autocorrelation

Spatial autocorrelation refers to similarities in attributes between adjacent locations compared to the attributes between more distant locations (Miller, 2004). Spatial autocorrelation in abundance data can be informative of processes that drive community patterns. Spatial autocorrelation in model residuals, however, can lead to incorrect interpretation of the processes that drive community patterns. To test for spatial autocorrelation within the sampled fracture water, MEM coefficients were calculated and those coefficients associated with positive Moran’s I were added to the GLM as independent variables. These coefficients may represent unknown processes occurring locally within the projected area. Local values for Moran’s I were also compared with a null distribution of the global Moran’s I to identify attributes at sampling locations (for example cell counts) with Moran’s I values that were more than two standard deviations from the null mean. A local Moran’s I for an attribute that is more than two standard deviations from the null mean in the positive direction indicates that the spatial distribution of that attribute is more clustered than would be expected if underlying spatial processes were random; in this case, the null hypothesis of random distribution of a given ‘attribute’ would be rejected. A local Moran’s I for an attribute that is more than two standard deviations from the null mean in the negative direction indicates the spatial distribution of high and low values for that attribute were more spatially dispersed than would be expected if underlying spatial processes were random; in this case, the null hypothesis would also be rejected.

**FIGURE 4:**
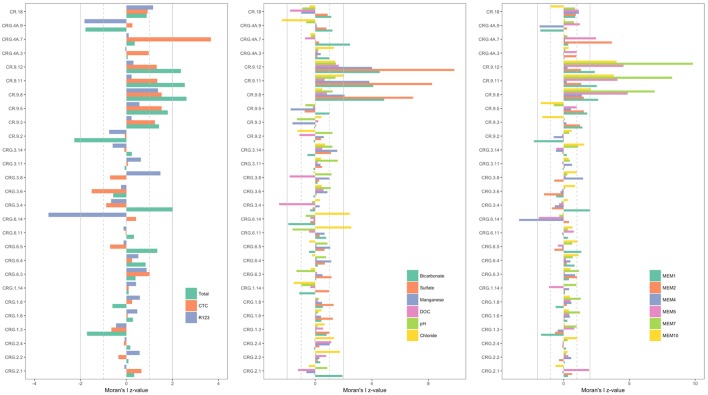
Spatial autocorrelation by sampling location [borehole and interval listed in a West-to-East direction starting with borehole CR-18 (see **Figure 1**)]. **(Left)**: total and viable cell densities. **(Middle)**: soluble components of the fracture water. **(Right)**: Positive Moran’s eigenvector map coefficients. The bars represent the *z*-values calculated by subtracting the local Moran’s I value from the null mean value and normalizing by the null standard deviation. Those locations with *z*-values more than two standard deviations from the null mean are significant. The gray lines mark first (dashed line) and second (solid line) standard deviations from the null mean.

The *z*-values calculated for the distribution of the various ‘attributes’ – namely, the cell count densities, concentrations of soluble compounds, pH and the positive MEM coefficients – are shown in **Figure 4**; the sampling locations are listed by borehole and interval following a West-to-East direction from borehole CR-18 to borehole CRG-2 (see **Figure 1**). The dashed gray lines and the solid gray lines mark where the first and second standard deviations from the null mean lie. The bars for attributes that extend beyond the mark for the second standard deviation, in the positive or negative direction, identify the sampling locations with spatially non-random attributes. From the plots in **Figure 4**, the deeper sampling locations within borehole CR-9 at intervals 8, 11, and 12, display non-random attributes relative to the global distribution of cell counts, or clustering as lower total cell counts; lower bicarbonate concentrations and higher sulfate and manganese concentrations; and by the MEM coefficients labeled MEM5, MEM7 and MEM10. These intervals are also the sampling locations with a saline signature (**Figures 2**, **3**, bottom left panel); even so, the chloride was not identified as being spatially autocorrelated.

The total cell counts from the shallow sampling location within the same borehole, located at interval 2, is dispersed compared to the null mean of spatially distributed counts values; and the MEM coefficient, MEM1. Only two other sampling locations had cell count densities that were outside the bounds of the null spatial distribution: interval 7 of borehole CRG-4A and interval 14 of borehole CRG-6. For these locations, interval 7 of borehole CRG-4A is elevated with respect to: the CTC positive counts; the concentration of bicarbonate; and the MEM coefficients, MEM2 and MEM5. Interval 14 of borehole CRG-6 is reduced with respect to: R123 positive counts; and the MEM coefficient MEM4. These deviations from the global Moran’s I for all the sampling locations suggest there are local processes that influencing the microbial abundances.

### Distribution of the Count Data

**FIGURE 5:**
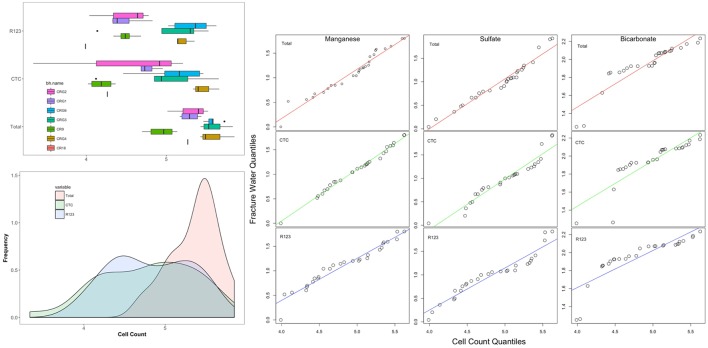
Distribution of total and viable cell counts. The boxplots are listed by borehole in a West-to-East direction starting with borehole CR-18 (see **Figure 1**). Also shown are quantile-by-quantile plots for cell density distributions compared to the distributions for fracture water manganese, sulfate and bicarbonate.

Values for total and viable cell densities in fracture water sampled from each borehole are provided in Supplementary Table S1. How the cell densities distribute across the sampling locations is shown in **Figure 5** as histograms and as boxplots by borehole arranged in a West to East direction (from borehole CR-18 to borehole CRG-2 as shown in **Figure 1**). The cell densities within boreholes CR-18, CR-9, CRG-1 and CRG-2 form the lower density part of the histograms and the cell densities within boreholes CRG-3, CRG-6 and CRG-4A form the higher density part of the histograms. The same data is plotted as scatter plots by sampling location elevation relative to sea level (Supplementary Figure S4, left panel) and by the Longitude value for the borehole collar, were the borehole enters the subsurface (Supplementary Figure S4, right panel; these two figures show that borehole location show a wider range of cell densities than does elevation.

To help identify possible drivers for the microbial abundances, the distribution patterns for the total and viable cell densities were also compared with the distributions of the fracture water geochemistry (using data taken from Supplementary Table S1) and to the rock porewater components: sulfate, bicarbonate, ammonia, nitrate and nitrite (from Peterman et al., 2016). The resulting quantile-by-quantile plots are shown in Supplementary Figures S5–S8 and in **Figure 5** beside the histograms for the cell densities. Quantile-by-quantile plots allow for the distribution patterns between two datasets to be compared; if the datasets follow a similar distribution the data points plot along a straight line; if the datasets do not follow a similar distribution pattern, the points diverge from a straight line. We find from these comparisons that the microbial cell densities distribute within the subsurface like that for the fracture water and porewater sulfate, the porewater ammonia and for the fracture water manganese, but not for the fracture water bicarbonate or for the porewater bicarbonate.

Supplementary Figure S5 show the cell density distributions with the fracture water and porewater sulfate. The total cell count data appear to have a distribution like that for the fracture water sulfate while the CTC and R123 cell density distributions deviate from the straight line (Supplementary Figure S5, top panel). The opposite patterns are seen for porewater sulfate; the distributions for total cell count and the porewater sulfate deviate from a straight line while the CTC and R123 cell densities appears to have distributions like that for the porewater sulfate (Supplementary Figure S5, bottom panel).

Comparisons with the distributions of fracture water and porewater bicarbonate (Supplementary Figure S6) and of fracture water manganese (Supplementary Figure S7) show that the count data distributions are not like that for bicarbonate in the lower quantiles (Supplementary Figure S6, top panel for fracture water, bottom panel for porewater) but they are distributed that for like manganese (Supplementary Figure S7).

Supplementary Figure S8 show the quantile-by-quantile plots for the distributions of cell density the porewater nitrogen compounds: ammonia, nitrite and nitrate. These components of the porewater were not detected within the fracture water. The plot shows that the distributions for total and CTC cell densities are roughly linear with the distribution for ammonia (Supplementary Figure S8, top panel). The total, CTC and R123 cell densities are also roughly linear with the distribution of nitrate concentrations but there is flattening in the middle quantiles for nitrate. The plot comparing the cell densities with porewater nitrite suggest these datasets follow different distributions across the formation (Supplementary Figure S8, bottom panel).

### Generalized Linear Modeling of the Count Data

The geochemical and descriptive data used for the GLM are given in Supplementary Table S1. The negative binomial GLM function within the R package ‘MASS’ (Venables and Ripley, 2002) provides a model to assign linear predictors (β) and a description of the random error distribution of the count data. The total and viable count data across all the sampling locations were each used as response variables. Data for the geochemical and positive MEM coefficients across all sampling locations were used as the dependent variables. Unmeasured environmental variables associated with the microbial cell density distribution would form part of the random component of the resulting linear model.

The independent variables were evaluated first for model selection then stepwise model fitting was performed. Metabolically relevant components of the geochemistry were: pH; dissolved organic carbon (DOC); bicarbonate; sulfate; iron; manganese; and phosphate. Bicarbonate ion is also a possible signature for modern recharge (**Figure 3**). Data for chloride ion were included as an explanatory variable for a saline source water component, and the stable oxygen isotope (δ^18^O) data were included as an explanatory variable for a glacial melt source water component. The spatial weightings matrix identified 11 positive MEM coefficients; these were also included in a model. The resulting coefficients (β) and the 5% confidence interval values for the significant explanatory variables are listed in Supplementary Table S2 (total counts), Supplementary Table S3 (CTC counts), Supplementary Table S4 (R123 counts) and Supplementary Table S5 (CFDA counts). Only those explanatory variables with a significance of *p* < 0.05 or lower are shown.

**FIGURE 6:**
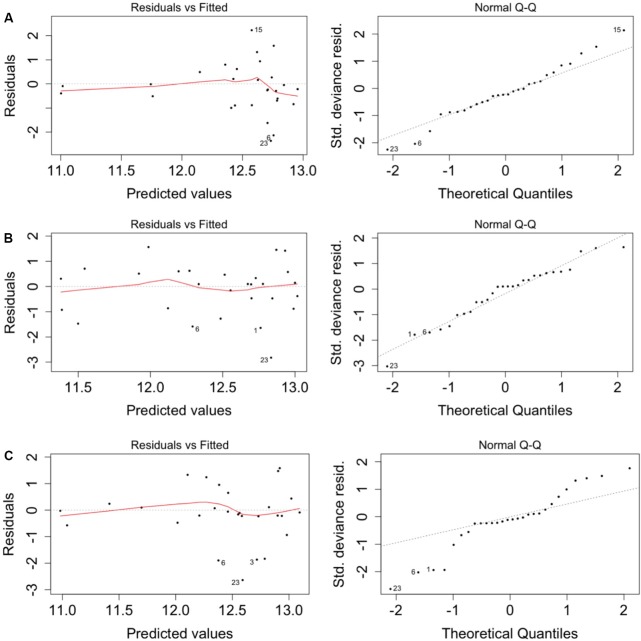
Spatial autocorrelation in the model residuals for the total cell count. **(A)** Significant environmental explanatory variables from Supplementary Table S1 (bicarbonate and manganese). **(B)** Significant spatial explanatory variables from Supplementary Table S1 (MEM1, MEM2, MEM4 and MEM5). **(C)** Significant environmental and spatial explanatory variables from Supplementary Table S1 (manganese, MEM1, MEM2 and MEM5).

When the models were run with the geochemistry – without the positive MEM coefficients – bicarbonate and manganese were identified as the predictors of total (Supplementary Table S2), CTC (Supplementary Table S3) and R123 (Supplementary Table S4) cell counts. When the model was run with the positive MEM coefficients – without the geochemistry – between two and four coefficients were significant: MEM2 and MEM4 were identified for both total and viable cell counts; and either MEM1, MEM5, MEM7 or MEM10 were identified depending on the count data (Supplementary Tables S2–S5). An analysis of the model residuals for the total count data are provided in **Figure 6**: environmental variables (**Figure 6A**), the positive MEM coefficients (**Figure 6B**) and the measured variables and spatial coefficients (**Figure 6C**). These plots show that the distribution of the residuals and fitted total count are more randomly distributed when the positive MEM coefficients are included in the model (**Figure 6B**) than when the model included only environment (**Figure 6A**). Combining spatial and environmental inputs did not improve the distribution of the model residuals (**Figure 6C**).

## Discussion

The concept of geological radioactive waste repositories is to provide secure locations over long residence times within deep geological settings allowing for the decay of long-lived radionuclides to background levels. The feasibility of emplacing a repository relies on having knowledge of local and regional subsurface dynamics that would form the basis of predicting the transport of hazardous species from a repository over the period of radioactive decay. Given the role of microbes on element speciation and transport and their effect on element retention by microbial-derived metal oxides (Kennedy et al., 2011), knowledge and understanding of local microbial ecology within prospective host formations can aid predictions used for making long term safety cases. To gauge interrelationships between subsurface microbial abundances with the geochemical data we combined the abundance data obtained from multiple sampling opportunities from within the crystalline formation underlying the Chalk River Laboratories site (Deep River, ON, Canada) and performed multivariate mixing and mass balance (M3) modeling, spatial analysis and GLM. We considered the dual role of fracture water – as the medium for transport of soluble species suitable for microbial metabolism and its role as the medium for dispersal.

In our analyses, we identified possible sources of the fracture water and evaluated the fracture water composition as predictors of total and viable microbial cell densities within these fractures including their distribution patterns. The dilute character of the fracture water (King-Sharp et al., 2016) compared to other sites on the Canadian Shield is thought to reflect recharge that occurred at the end of the last glaciation followed by a gradual recharge with meteoric water. The fracture water ages date from 5000 to 10,000 years (King-Sharp et al., 2016). Modern recharge does not appear to extend deeper than approximately 100 m (King-Sharp et al., 2016). We therefore performed modeling to address whether recharge can explain how subsurface communities assemble within these fractures. The main findings from M3 modeling were that three possible meteoric source waters account for 97% of the samples: glacial melt water, a saline source and modern recharge. The mixing proportion for modern recharge and glacial melt water describe most of the samples; the mixing proportion of a saline source water is localized to deeper fractures transected by boreholes CR-9 (**Figure 3**).

Although the stable isotope data for oxygen and hydrogen align with the VSMOW (Supplementary Figure S1), supporting the notion of recharge as a main driver of microbial assembly, rock water interactions may still be important in explaining the fracture water microbiology. A porewater analysis of the drilled rock cores identified nitrogen compounds (Peterman et al., 2016) that were not detected within the fracture water; a finding that corroborates both the measured nitrogen metabolism within the fracture water (Stroes-Gascoyne et al., 2011) and the identified taxa within the fracture water (Beaton et al., 2016) whose cultured relatives encompass the complete nitrogen cycle, including nitrogen fixation. In a study of the component taxa (manuscript in preparation), nitrogen metabolism was detected within all sampling locations; sulfate reduction was detected only within borehole CRG-6.

Recharge into fractures is topology driven. An analysis of the cell density distribution patterns identified location specific patterns and patterns that were generalized across the sampling locations. The total and viable cell densities fell into two categories: those locations with lower cell densities (location within boreholes CR-9, CR-18, CRG-1, CRG-2) (**Figure 5** and Supplementary Figure S4) and those locations with higher cell densities (locations within boreholes CRG-3, CRG-4A and CRG-6). The sampling locations with the lowest total cell counts were those locations that had the highest mixing proportions of a saline source water (**Figure 2**) and the sampling locations with the highest total cell densities were those locations with higher mixing proportions of modern recharge (**Figure 2**). Variation in the count data appears to be localized to the region around each of the boreholes and not to the elevation of the sampling locations (Supplementary Figure S4). If the abundance data can be linked to recharge, the influences of local conditions on recharge may need to be accounted for by, for example, overburden thickness and hydraulic conductivity. In a study of modern recharge into another fractured crystalline aquifer that is overlain by variable thicknesses of overburden (Gleeson et al., 2009), the authors conclude that overburden thickness and hydraulic conductivity were major parameters that controlled modern recharge into the underlying bedrock aquifer and that a thicker overburden meant modern recharge was slower and more widespread (Gleeson et al., 2009). A slower recharge rate and higher surface area of unconsolidated overburden would favor higher cell densities.

The distributions of the abundances and the geochemistry were more generalized across the site. The quantile-by-quantile plots show that the total cell count distributions aligned with the distributions of fracture water sulfate, fracture water manganese, porewater sulfate, porewater ammonia and porewater nitrate (**Figure 5** and Supplementary Figures S5, S7, S8). The viable cell count distributions also aligned with the distributions of sulfate, manganese, ammonia and nitrate while their distributions compared with bicarbonate were heavy tailed in the lower quantiles – namely, those sampling locations within borehole CR-9; a region of the subsurface found to be distinct in the M3 modeling (**Figure 3**).

Analysis of spatially distributed sampling locations can reveal distance-relationships in abundance data (Dormann et al., 2007). The assumptions made when modeling population abundances can lead to incorrect conclusions if the model residuals are not randomly distributed (Dormann et al., 2007). We therefore performed a spatial analysis to test for a role for meteoric water recharge on total and viable abundances by comparing a null distribution of the global Moran’s I value (**Figure 4**) and by adding the resulting eigenvector map coefficients into a GLM (**Figure 6**). The GLM identified bicarbonate and manganese as significant predictors of microbial abundances (Supplementary Tables S2–S5). Both bicarbonate and manganese also show spatial autocorrelation at sampling locations within borehole CR-9; as does sulfate (**Figure 4**). In our analysis, bicarbonate was considered as a proxy for modern recharge; the proxy for a saline source recharge, chloride, was not identified as significant (Supplementary Tables S2–S5) and, despite the M3 modeling showing the localization of this saline signature (**Figures 2**, **3**), chloride was not spatially clustered with the bicarbonate, manganese and sulfate (**Figure 4**).

The GLM also identified positive MEM coefficients of which four coefficients clustered within borehole CR-9 and two coefficients, MEM2 and MEM4, were randomly distributed. The improved GLM residuals with these coefficients suggest that the significance of the bicarbonate and manganese was due to the localized and distinct fracture water conditions that exist within borehole CR-9 and further suggests that their significance in the GLM reflects this spatial correlation. Inclusion of the MEM coefficients within the GLM improved the distribution of the GLM residuals. The finer scale influences represented by these coefficients may indicate unmeasured/unknown processes; the distribution pattern similarities observed with the quantile-by-quantile plots may help reconcile these processes.

## Conclusion

The main findings of this work are that M3 modeling identified three possible meteoric sources water for recharge; of these three, modern recharge appears to be the most likely source water to explain, in part, microbial abundances within the projected area of the sampling locations. Chloride, as a proxy for a saline source water, was not a significant explanatory variable for the total or viable count data. Stable oxygen isotope (δ^18^O), as a gauge of glacial melt water, was also not a significant explanatory variable of microbial abundance distributions.

Spatial autocorrelation analysis show that low total cell counts co-localize with lower bicarbonate, higher manganese and higher sulfate. These locations are associated with the saline source water signatures. The spatial correlation of both the bicarbonate and the manganese suggest that their significance in the GLM reflects this spatial correlation and not a direct effect on microbial abundances, *per se*. Inclusion of positive MEM coefficients into the GLM improved the distribution of the model residuals. The finer scale influences represented by the significant MEM coefficients suggest there are unmeasured/unknown processes occurring within these sampling locations.

While the fracture water is dilute, and of mainly meteoric origin (King-Sharp et al., 2016), the prospect of porewater sulfur and porewater nitrogen (Peterman et al., 2016) potentially leaching from the host rock suggest there may be localized processes that are separate from a role of source water recharge in explaining microbial abundance distributions within the projected area of the sampling locations.

## Author Contributions

All authors collected and contributed data sets for analysis as well as participated in the conceptual drafting and revision of this manuscript. KK-S oversaw the groundwater sampling. IG performed modelling of the geochemistry. EB conducted subsequent analyses and was the primary author in writing and revising the manuscript. SS-G, KK-S, and MS contributed significantly in manuscript development and revision.

## Conflict of Interest Statement

The authors declare that the research was conducted in the absence of any commercial or financial relationships that could be construed as a potential conflict of interest.

## References

[B1] Beaton E. D. Stevenson B. S. King-Sharp K. J. Stamps B. W. Nunn H. S. Stuart M. (2016). Local and regional diversity reveals dispersal limitation and drift as drivers for groundwater bacterial communities from a fractured granite formation. *Front. Microbiol.* 7:1933 10.3389/fmicb.2016.01933 PMC5138202 27999569

[B2] Bliss C. I. Fisher R. A. (1953). Fitting the negative binomial distribution to biological data. *Biometrics* 9 176–200. 10.2307/3001850

[B3] Borcard D. Legendre P. (2002). All-scale spatial analysis of ecological data by means of principal coordinates of neighbour matrices. *Ecol. Model.* 153 51–68. 10.1016/S0304-3800(01)00501-4

[B4] Dormann C. F. McPherson J. Araújo M. B. Bivand R. Bolliger J. Carl G. (2007). Methods to account for spatial autocorrelation in the analysis of species distributional data: a review. *Ecography* 30 609–628. 10.1111/j.2007.0906-7590.05171.x

[B5] Dray S. Legendre P. Peres-Neto P. R. (2006). Spatial modelling: a comprehensive framework for principal coordinate analysis of neighbour matrices (PCNM). *Ecol. Model.* 196 483–493. 10.1016/j.ecolmodel.2006.02.015

[B6] El-Shaarawi A. H. Esterby S. R. Dutka B. J. (1981). Bacterial density in water determined by Poisson or negative binomial distributions. *Appl. Environ. Microbiol.* 41 107–116. 16345678 10.1128/aem.41.1.107-116.1981 PMC243648

[B7] Frape S. K. Fritz P. (eds) (1987). “Geochemical trends from groundwaters from the Canadian Shield,” in *saline Water and Gases in Crystalline Rocks, Special Paper 33* (St. John’s, NL: Geological Association of Canada), 19–38.

[B8] Fuller M. E. Streger S. H. Rothmel R. K. Mailloux B. J. Hall J. A. Onstott T. C. (2000). Development of a vital fluorescent staining method for monitoring bacterial transport in subsurface environments. *Appl. Environ. Microbiol.* 66 4486–4496. 10.1128/AEM.66.10.4486-4496.2000 11010903 PMC92329

[B9] Gilbert B. Bennet J. R. (2010). Partitioning variation in ecological communities: do the numbers add up? *J. Appl. Ecol.* 47 1071–1082. 10.1111/j.1365-2664.2010.01861.x

[B10] Gleeson T. Novakowski K. Kurt Kyser T. (2009). Extremely rapid and localized recharge to a fractured rock aquifer. *J. Hydrol.* 376 496–509. 10.1016/j.jhydrol.2009.07.056

[B11] Haas C. N. Heller B. (1986). Statistics of enumerating total coliforms in water samples by membrane filter procedures. *Water Res.* 20 525–530. 10.1016/0043-1354(86)90203-4

[B12] Harrison X. A. (2014). Using observation-level random effects to model overdispersion in count data in ecology and evolution. *PeerJ* 2:e616 10.7717/peerj.616 PMC4194460 25320683

[B13] Haveman S. A. Pedersen K. Ruotsalainen P. (1999). Distribution and metabolic diversity of microorganisms in deep igneous rock aquifers of Finland. *Geomicrobiol. J.* 16 277–294. 10.1080/014904599270541

[B14] Hilbe J. M. (2011). *Negative Binomial Regression*, Second Edn Cambridge: Cambridge University Press 10.1017/CBO9780511973420

[B15] Hubbell S. P. (2001). *The Unified Neutral Theory of Biodiversity and Biogeography.* Princeton, NJ: Princeton University Press.

[B16] Jain D. K. Providenti M. Tanner C. Cord I. Stroes-Gascoyne S. (1997). Characterization of microbial communities in deep groundwater from granitic rock. *Can. J. Microbiol* 43 272–283. 10.1139/m97-038

[B17] Kennedy C. B. Gault A. G. Fortin D. Clark I. D. F. G. Ferris F. G. (2011). Retention of iodide by bacteriogenic iron oxides. *Geomicrobiol. J.* 28 387–395. 10.1080/01490451003653110

[B18] Kieft T. L. (1990). *Environmental Parameters Controlling Microbial Activities in Terrestrial Subsurface Environments.* Washington, DC: US Department of Energy.

[B19] King-Sharp K. Frape S. K. Peterman Z. E. Gwynne R. Tian L. Gurban I. (2016). Synthesis of geochemical and fracture mineral studies relevant to a deep geological repository for non-fuel wastes at Chalk River. *CNL Nuclear Rev.* 6 117–130. 10.12943/CNR.2016.00015

[B20] Laaksoharju M. Gascoyne M. Gurban I. (2008). Understanding groundwater chemistry using mixing models. *Appl. Geochem.* 23 1921–1940. 10.1016/j.apgeochem.2008.02.018

[B21] Laaksoharju M. Skårman C. Skårman E. (1999). Multivariate mixing and mass balance (M3) calculations, a new tool for decoding hydrogeochemical information. *Appl. Geochem.* 14 861–871. 10.1016/S0883-2927(99)00024-4

[B22] McFeters G. A. Pyle B. H. Lisle J. T. Broadaway S. C. (1998). Rapid direct methods for enumeration of specific, active bacteria in water and biofilms. *J. Appl. Microbiol.* 85 193S–200S. 10.1111/j.1365-2672.1998.tb05299.x 21182709

[B23] Miller H. J. (2004). Tober’s first law and spatial analysis. *Ann. Assoc. Am. Geogr.* 94 284–289. 10.1111/j.1467-8306.2004.09402005.x

[B24] Muyzer G. de Waal E. C. Uitterlinden A. G. (1993). Profiling of complex microbial populations by denaturing gradient gel electrophoresis analysis of polymerase chain reaction-amplified genes coding for 16S rRNA. *Appl. Environm. Microbiol.* 59 695–700. 10.1128/aem.59.3.695-700.1993 PMC202176 7683183

[B25] Nemergut D. R. Schmidt S. K. Fukami T. O’Neill S. P. Bilinski T. M. Stanish L. F. (2013). Patterns and processes of microbial community assembly. *Microbiol. Mol. Biol. Rev.* 7 342–356. 10.1128/MMBR.00051-12 PMC3811611 24006468

[B26] Nyyssönen M. Bomberg M. Kapanen A. Nousiainen A. Pitkänen P. Itävaara M. (2012). Methanogenic and sulphate-reducing microbial communities in deep groundwater of crystalline rock fractures in Olkiluoto, Finland. *Geomicrobiol. J.* 29 863–878. 10.1080/01490451.2011.635759

[B27] Peterman Z. E. Neymark L. A. King-Sharp K. J. Gascoyne M. (2016). Isotope hydrology of the Chalk River Laboratories site, Ontario, Canada. *Appl. Geochem.* 66 149–161. 10.1016/j.jenvrad.2014.07.007

[B28] Remenda V. H. Cherry J. A. Edwards T. W. (1994). Isotopic composition of old ground water from Lake Agassiz: implications for late Pleistocene climate. *Science* 266 1975–1978. 10.1126/science.266.5193.1975 17836515

[B29] Rozanski K. Araguás-Araguás L. Gonfiantini R. (1993). “Isotopic patterns in modern global precipitation,” in *Climate Change in Continental Isotopic Records*, eds Swart P. K. Lohmann K. C. McKenzie J. Savin S. (Washington, DC: American Geophysical Union).

[B30] Sahl J. W. Schmidt R. Swanner E. D. Mandernack K. W. Templeton A. S. Kieft T. L. (2008). Subsurface microbial diversity in deep-granitic-fracture water in Colorado. *Appl. Environ. Microbiol.* 74 143–152. 10.1128/AEM.01133-07 17981950 PMC2223202

[B31] Schaule G. Flemming H. C. Ridgway H. F. (1993). Use of 5-cyano-2,3-ditolyl tetrazolium chloride for quantifying planktonic and sessile respiring bacteria in drinking water. *Appl. Environ. Microbiol.* 59 3850–3857. 8285688 10.1128/aem.59.11.3850-3857.1993 PMC182540

[B32] Stroes-Gascoyne S. Hamon C. J. Audette-Stuart M. Beaton E. D. King-Sharp K. Festarini A. (2011). *Microbial Characterization of Groundwater from Boreholes CR9 and CR18 at CRL (2007–2009) – Implications for a Possible Future Repository for Radioactive Non-fuel Waste.* Toronto, ON: Canadian Nuclear Society.

[B33] Thompson P. Baumgartner P. Beaton E. D. Chan T. Kitson C. Kozak E. (2011). *An Investigation of the Suitability of the Chalk River Site to Host a Geologic Waste Management Facility for AECL’s Low and Intermediate Level Wastes.* Toronto, ON: Canadian Nuclear Society.

[B34] Torrance J. K. (1988). “Mineralogy, pore-water chemistry and geotechnical behaviour of Champlain Sea and related sediments,” in *The Late Quaternary Development of the Champlain Sea Basin*, ed. Gadd N. R. (St. John’s, NL: Geological association of Canada).

[B35] Vellend M. (2010). Conceptual synthesis in community ecology. *Q. Rev. Biol.* 85 183–206. 10.1086/652373 20565040

[B36] Venables W. N. Ripley B. D. (2002). *odern Applied Statistics with S.* New York, NY: Springer Science Business Media.

