# Supplementary material for: Spatial Autocorrelation, Source Water and the Distribution of Total and Viable Microbial Abundances within a Crystalline Formation to a Depth of 800 m
Source: Front Microbiol. 2017 Sep 19;8:1731. doi: 10.3389/fmicb.2017.01731 (PMC5610697; doi:10.3389/fmicb.2017.01731)
Supplement: Supplementary file 2 [file Image_1.pdf]

Supplemental Information

Table S1. The Geochemical and Cell Count Data Used for Modelling Total and Viable Microbial Cell Densities

Borehole and Interval	*	Easting	Northing	Elevation	pH	Dissolved Organic Carbon	Bicarbonate	Sulfate	Chloride	Iron	Manganese	Total Cell Count	CTC Count	R123 Count	CFDA Count
				m		mg/L	mg/L	mg/L	mg/L	µg/L	µg/L	cells/mL	cells/mL	cells/mL	cells/mL
CRG.1.3	2	313743.6	5102838	31.3	8.5	13.4	133.3	1.3	8.2	42	28.5	1.60×10^{5}	4.80×10^{4}	2.73×10^{4}	1.56×10^{4}
CRG.1.6	2	313760.8	5102857	-67.7	8.4	12.5	120.1	0.6	34.5	48.5	38.0	2.36×10^{5}	4.84×10^{4}	2.16×10^{4}	4.41×10^{3}
CRG.1.8	2	313771.9	5102870	-130.7	8.6	12.1	118.1	0.1	9.0	84.5	25.9	1.47×10^{5}	5.96×10^{4}	2.11×10^{4}	7.11×10^{3}
CRG.1.14	2	313815.2	5102917	-355.7	9.1	6.5	70.8	3.6	58.0	22	12.9	2.74×10^{5}	9.02×10^{4}	6.73×10^{4}	3.43×10^{4}
CRG.2.1	1	314741	5104127.2	137.9	7.8	1.2	152.4	19.0	1.8	29	37.0	3.32×10^{5}	1.61×10^{5}	1.09×10^{4}	2.68×10^{3}
CRG.2.2	2	314757.5	5104142	92.2	8.6	23.0	116.8	11.4	0.5	20	10.0	1.03×10^{5}	2.17×10^{3}	6.02×10^{4}	2.01×10^{3}
CRG.2.4	1	314777	5104160	38.2	9.4	17.4	83.1	10.8	1.0	100	0.0	2.52×10^{5}	8.22×10^{4}	4.36×10^{4}	3.68×10^{3}
CRG.3.4	1	313346.9	5102436.2	-60.3	9.5	3.0	84.0	21.2	7.5	20	6.5	4.69×10^{5}	1.32×10^{4}	1.37×10^{4}	5.18×10^{3}
CRG.3.6	1	313344.3	5102404	-122.0	9.2	33.0	90.0	16.0	12.5	49	3.0	6.76×10^{5}	4.52×10^{5}	3.36×10^{5}	2.04×10^{5}
CRG.3.8	2	313335.7	5102336	-247.0	9.2	1.5	116.6	11.0	56.0	14	2.6	2.67×10^{5}	7.09×10^{4}	1.99×10^{5}	3.08×10^{4}
CRG.3.11	2	313318.3	5102241	-417.0	9.3	1.6	83.3	9.0	21.5	145.5	14.9	3.38×10^{5}	1.85×10^{5}	2.20×10^{5}	2.40×10^{4}
CRG.3.14	2	313293.2	5102122	-617.0	9.5	1.2	78.9	2.2	21.9	70.5	2.3	2.95×10^{5}	8.66×10^{4}	8.76×10^{4}	1.17×10^{4}
CRG.4A.3	1	312209.8	5104600.7	1.8	8.6	24.7	122.5	14.8	2.0	30	10.0	2.65×10^{5}	2.50×10^{5}	1.36×10^{5}	6.23×10^{4}
CRG.4A.7	1	312208	5104495.3	-310.9	8.8	2.5	147.4	5.2	4.9	0	17.0	3.08×10^{5}	4.58×10^{5}	1.39×10^{5}	2.66×10^{4}
CRG.4A.9	1	312203.4	5104452	-440.0	7.9	18.4	124.3	2.0	14.0	50	20.0	7.10×10^{5}	2.15×10^{5}	2.25×10^{5}	2.85×10^{4}
CRG.4A.13	1	312178.3	5104338	-799.2	7.3	51.0	463.2	0.3	398.3	1900	630.0	9.74×10^{5}	2.90×10^{5}	4.28×10^{5}	1.31×10^{5}
CRG.6.3	2	313391.2	5102135	48.0	8.4	9.5	110.0	3.6	6.0	47	14.0	3.29×10^{5}	2.90×10^{5}	2.53×10^{5}	1.66×10^{4}
CRG.6.4	2	313373.8	5102116	-4.0	9.1	9.9	91.0	5.5	15.0	68	4.0	3.87×10^{5}	2.09×10^{5}	2.11×10^{5}	2.74×10^{4}
CRG.6.5	1	313351.6	5102092	-68.0	9.0	10.4	74.0	4.7	21.0	430	5.0	5.32×10^{5}	2.88×10^{4}	1.05×10^{5}	6.97×10^{3}
CRG.6.9	1	313294.5	5102036.1	-229.0	8.7	1.9	170.2	7.1	33.0	73	15.0	3.81×10^{5}	2.80×10^{5}	4.22×10^{5}	5.77×10^{4}
CRG.6.11	1	313234.8	5101980	-390.0	8.0	12.9	116.0	10.4	32.0	178	6.0	3.69×10^{5}	9.36×10^{4}	1.08×10^{5}	1.79×10^{4}
CRG.6.14	1	313203.3	5101951.7	-469.0	9.0	1.8	131.0	11.4	32.2	20	16.0	2.90×10^{5}	1.01×10^{5}	3.39×10^{5}	3.60×10^{4}
CR.9.2	2	312721.5	5102262	29.0	9.0	28.5	120.6	6.4	35.0	79	12.0	8.62×10^{4}	2.32×10^{4}	4.82×10^{4}	1.91×10^{4}
CR.9.3	2	312691.7	5102245.9	-26.2	8.1	19.1	69.0	17.0	137.7	47.7	42.8	1.25×10^{5}	1.64×10^{4}	3.09×10^{4}	4.18×10^{3}
CR.9.5	4	312617.4	5102207	-154.0	9.0	12.7	83.8	9.4	43.3	19.8	3.7	1.37×10^{5}	1.44×10^{4}	3.04×10^{4}	5.09×10^{3}
CR.9.8	4	312466.6	5102145	-364.0	8.2	5.0	41.6	53.0	152.6	51.5	35.0	9.94×10^{4}	2.18×10^{4}	3.58×10^{4}	8.49×10^{3}
CR.9.11	4	312449.7	5102130.9	-385.0	7.7	6.4	17.3	79.3	1422.5	21	61.8	5.82×10^{4}	1.05×10^{4}	2.60×10^{4}	1.03×10^{4}
CR.9.12	2	312416.1	5102120.0	-426.3	7.9	2.1	16.9	82.8	1665.8	3.3	62.3	4.96×10^{4}	1.09×10^{4}	2.18×10^{4}	9.24×10^{3}
CR.18	4	311262.7	5103333	111.0	9.0	1.3	70.4	25.0	95.5	39.5	6.0	1.85×10^{5}	1.83×10^{4}	9.75×10^{3}	2.34×10^{3}

* Number of sampling days. On each sampling day, three samplings were performed. Total direct counts for each sampling were performed in triplicate. At least 15 fields of view and at least 300 cells counted per filter to give a maximum error of 5.8% per filter. Viable counts were performed on one of the samplings, also in triplicate. Coefficients of variation for the triplicate determinations ranged from 1-70% for the total count data and between 10-150% for the viable count data.

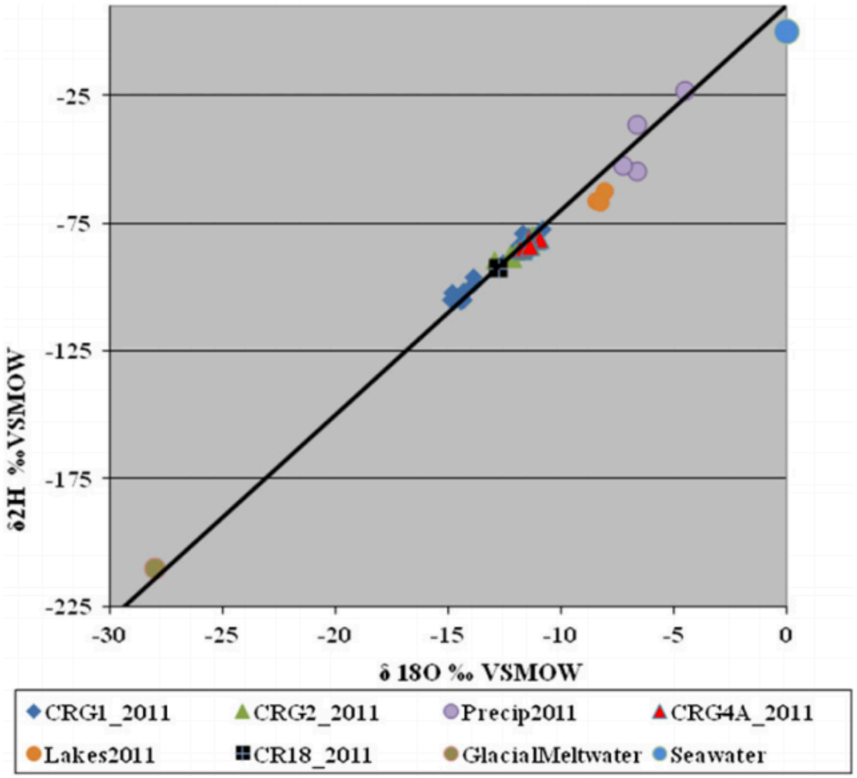

Figure S1. Stable oxygen and stable hydrogen isotopes of dilute fracture groundwater compared to the Vienna Standard Mean Ocean Water (black solid line). From King-Sharp, 2016.

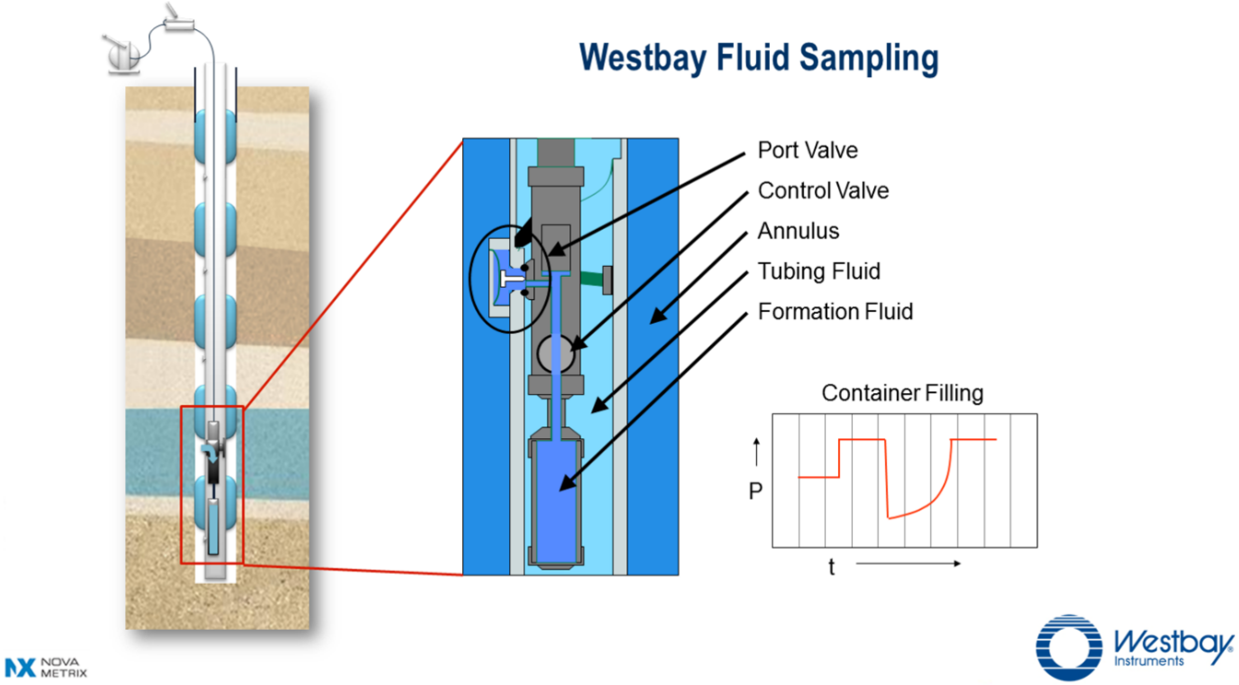

Figure S2. Schematic of the Westbay System.

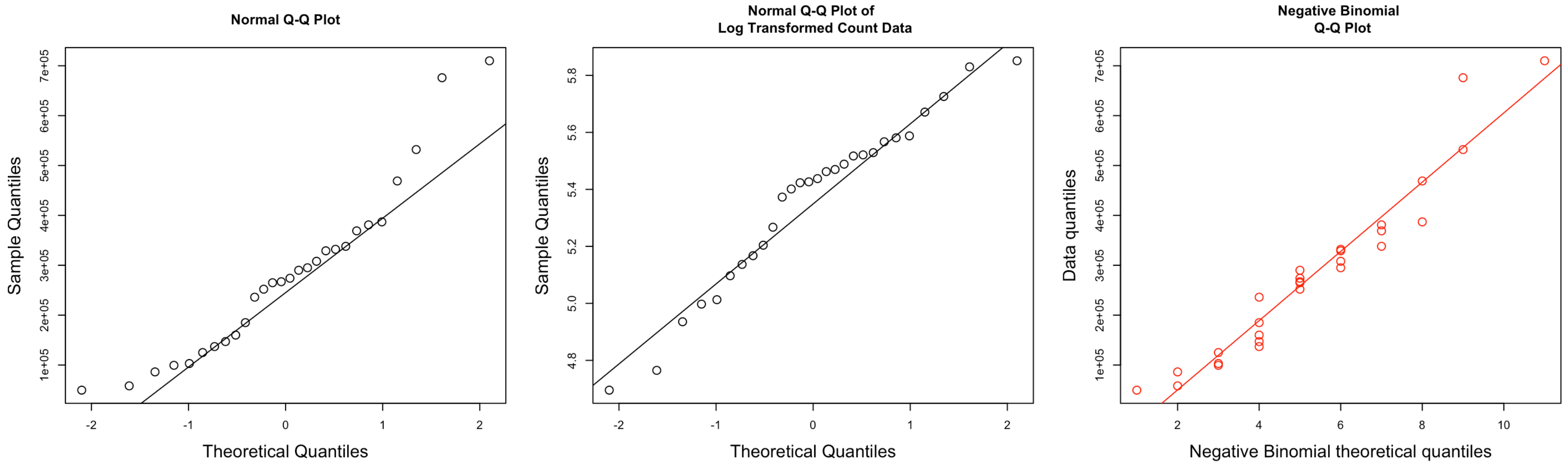

Figure S3. Distribution of the subsurface total cell abundance with a theoretical normal distribution (left and middle panel) and a negative binomial distribution (right panel)

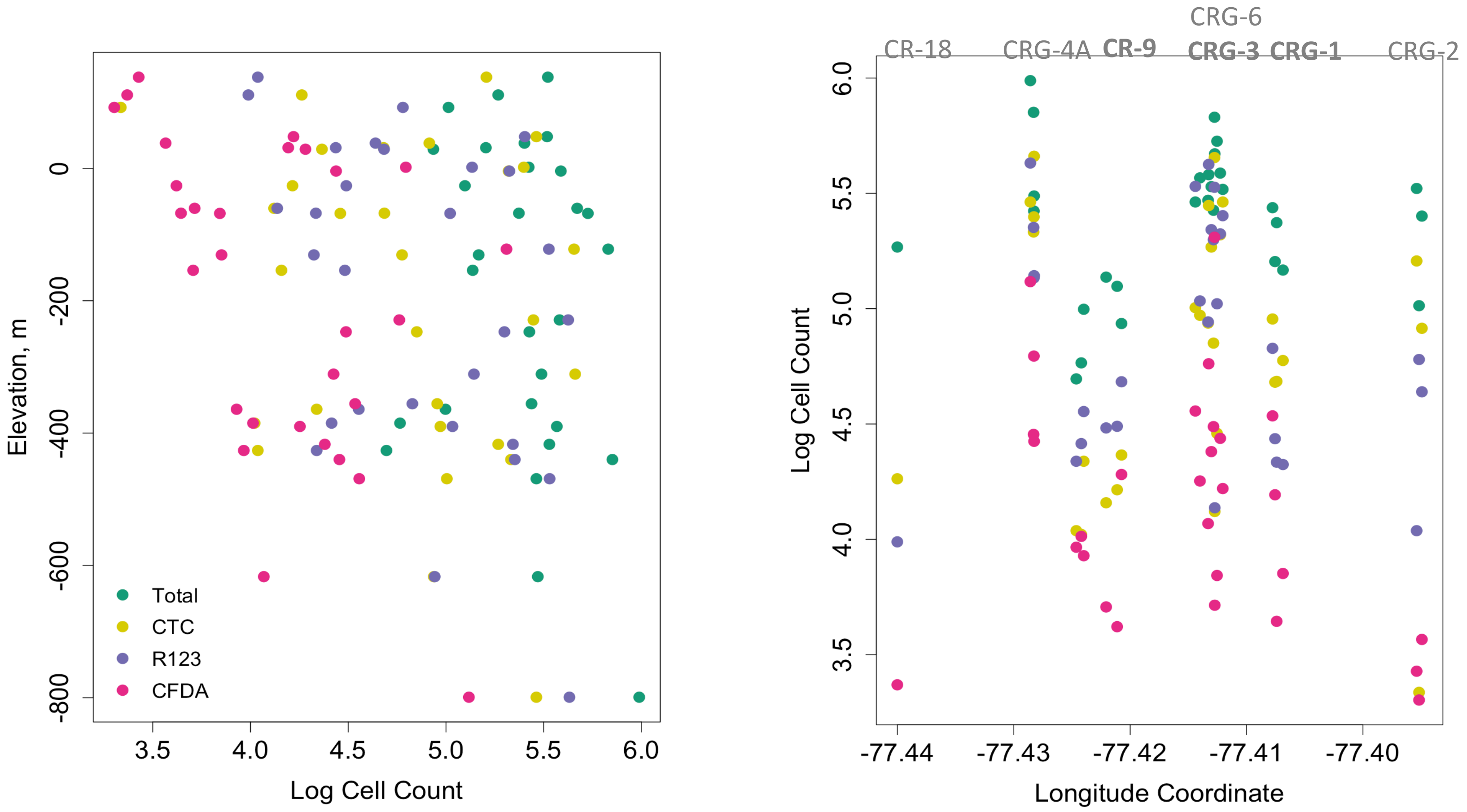

Figure S4 The distributions of total and viable cell densities by elevation and by the location of the borehole collar, shown here as the Longitude coordinate for each of the boreholes.

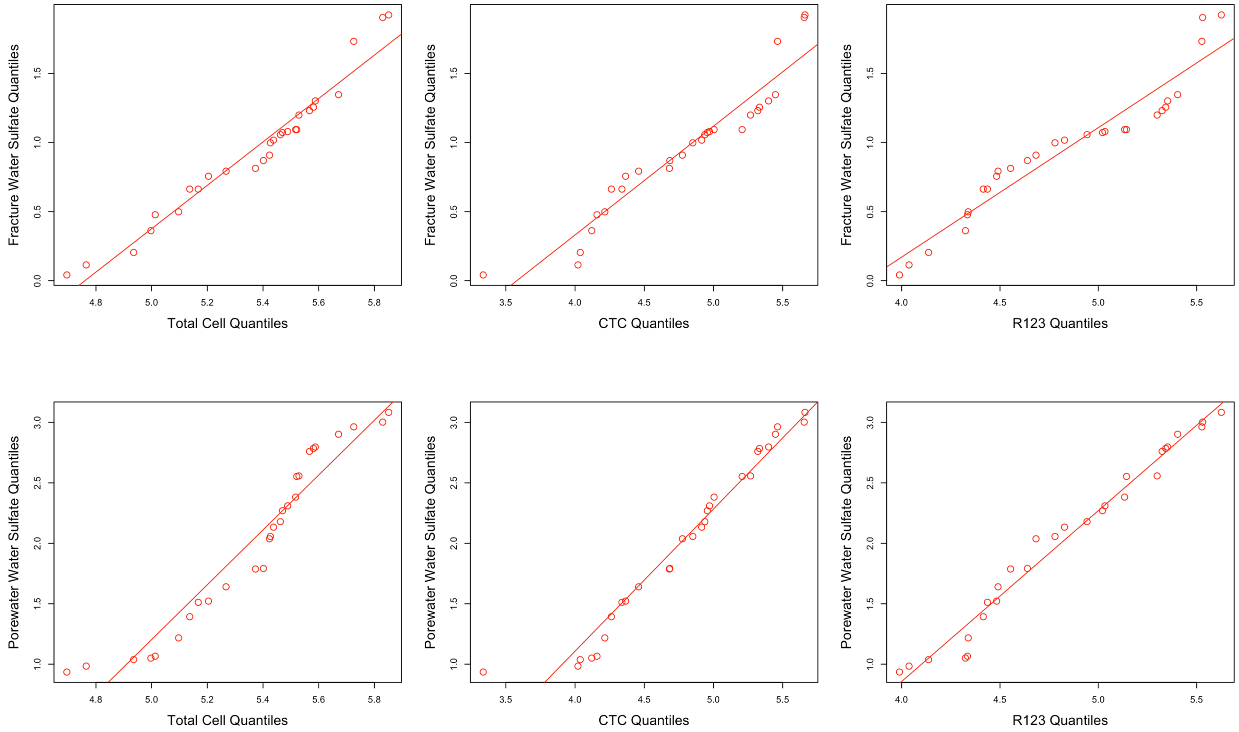

Figure S5 Sulfate: Quantile-by-quantile plots (qqplot). Distribution of sulfate in fracture water (top panel) and porewater water (bottom panel) with the distributions of the total and viable microbial abundances. The data were log transformed for the comparison. The data for porewater was taken from Peterman et al. (2016), and the data for fracture water was taken from Table S1.

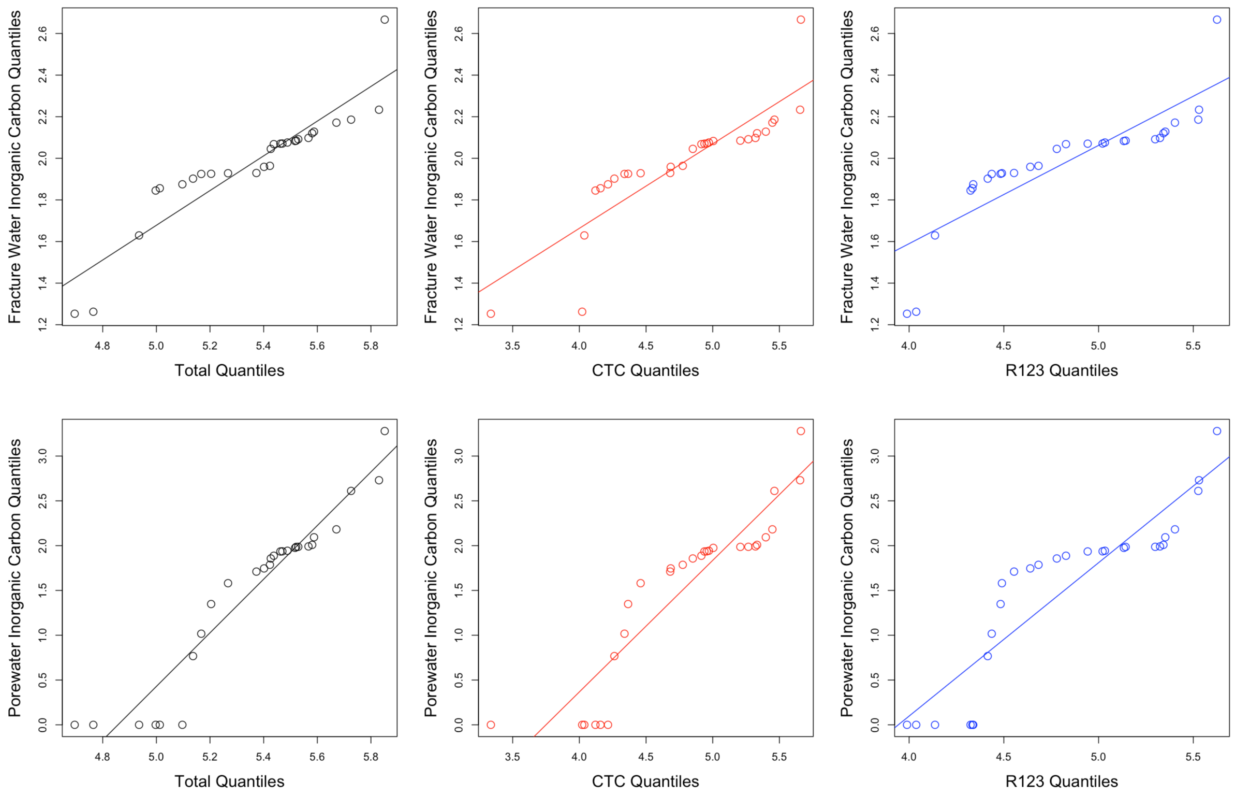

Figure S6 Inorganic carbon: Quantile-by-quantile plots (qqplot). Distribution of inorganic carbon in fracture water (top panel) and porewater water (bottom panel) with the distributions of the total and viable microbial abundances. The data were log transformed for the comparison. The data for porewater was taken from Peterman et al. (2016), and the data for fracture water was taken from Table S1.

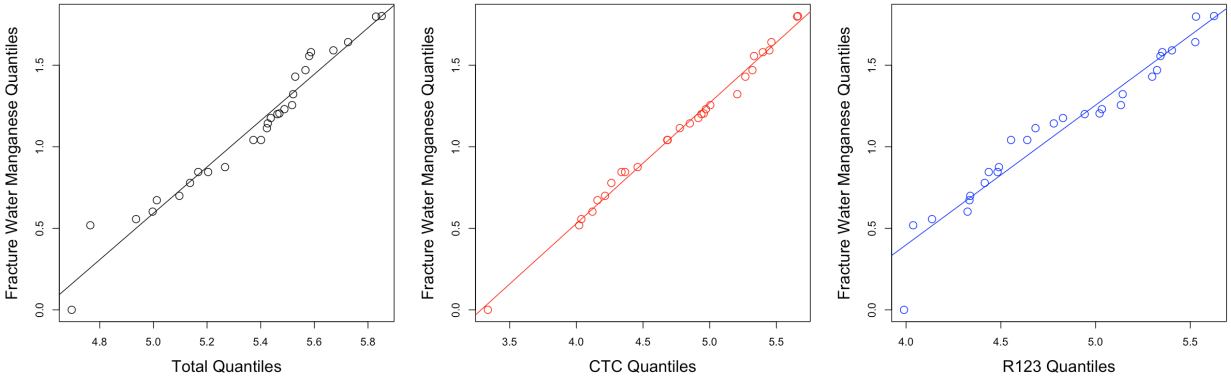

Figure S7 Manganese: Quantile-by-quantile plots (qqplot). Distribution of manganese in fracture water with the distributions of the total and viable microbial abundances. The data were log transformed for the comparison. The data for fracture water was taken from Table S1.

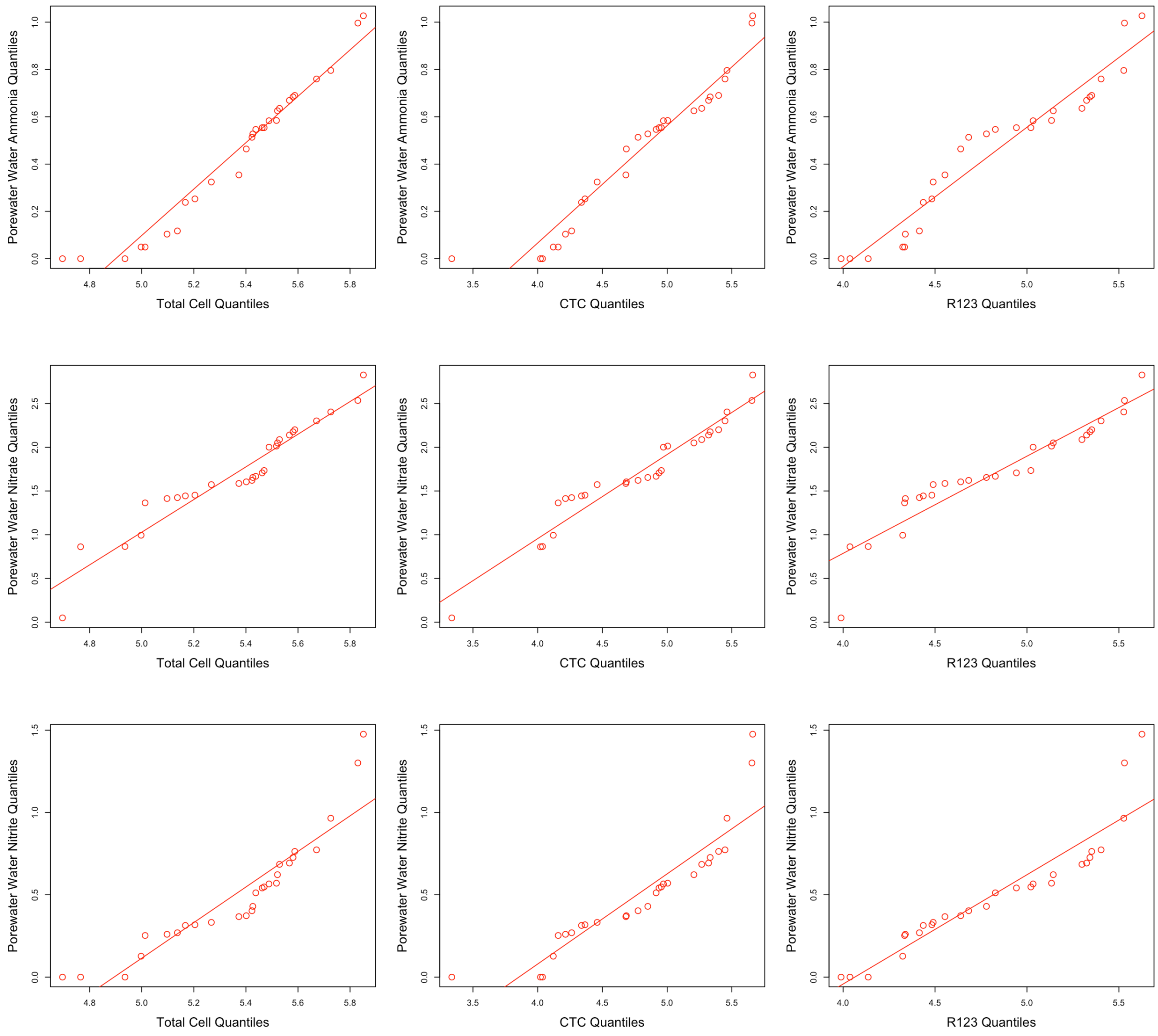

Figure S8 Ammonia, nitrate and nitrite: Quantile-by-quantile plot (qqplot) comparing the distribution of ammonia (top panel), nitrate (middle panel) and nitrite (bottom panel) in porewater with the distributions of the total and viable microbial abundances. The data were log transformed for the comparison. The data for porewater was taken from Peterman et.al. (2016).

Table S2. Total Microbial Cell Count Data: Coefficients and 5% Confidence Intervals from the Negative Binomial GLM for the Correlation with the Explanatory Variables: Environment and Space.

	Coefficient	Confidence Interval		Significance
		2.5%	97.5%	
**Environment: theta = 4.98 ± 1.29**				
(Intercept)	12.3	11.7	12.9	
Bicarbonate	5.8×10^{-3}	7.4×10^{-4}	1.2×10^{-2}	*
Manganese	-2.2×10^{-2}	-3.3×10^{-2}	-1.2×10^{-2}	***
**Spatial: theta = 5.76 ± 1.47**				
(Intercept)	12.4	12.3	12.6	
MEM1	0.181	0.028	0.335	*
MEM2	0.387	0.231	0.544	***
MEM4	-0.176	-0.346	0.000	*
MEM5	-0.198	-0.349	-0.046	*
**Environment + Spatial: theta = 6.70 ± 1.75**				
(Intercept)	12.7	12.5	12.97	
manganese	-0.02	-0.03	-0.01	**
MEM1	0.18	0.03	0.32	*
MEM2	0.26	0.09	0.43	**
MEM5	-0.22	-0.36	-0.08	**

* p<0.05,

** p<0.01

*** p<0.001

Table S3. CTC Microbial Cell Count Data: Coefficients and 5% Confidence Intervals from the Negative Binomial GLM for the Correlation with the Explanatory Variables: Environment and Space.

	Coefficient	Confidence Interval		Significance
		2.5%	97.5%	
**Environment: theta = 1.26 ± 0.30**				
(Intercept)	10.4	9.2	11.6	
Bicarbonate	1.6×10^{-2}	0.6×10^{-2}	2.6×10^{-2}	***
Manganese	-2.5×10^{-2}	-4.6×10^{-2}	-0.3×10^{-2}	*
**Spatial: theta = 1.47 ± 0.36**				
(Intercept)	11.4	11.1	11.7	
MEM1	0.44	0.15	0.72	**
MEM2	0.57	0.27	0.88	***
MEM4	-0.30	-0.63	0.04	*
MEM10	-0.33	-0.66	-0.01	*
**Environment + Spatial: theta = 1.52 ± 0.37**				
(Intercept)	10.4	9.4	11.4	
MEM2	0.46	0.15	0.79	**
MEM4	-0.33	-0.67	0.03	*

* $p<0.05$,

** $p<0.01$

*** $p<0.001$

Table S4. R123 Microbial Cell Count Data: Coefficients and 5% Confidence Intervals from the Negative Binomial GLM for the Correlation with the Explanatory Variables: Environment and Space.

	Coefficient	Confidence Interval		Significance
		2.5%	97.5%	
**Environment: theta = 1.62 ± 0.40**				
(Intercept)	11.4	10.0	12.5	
Bicarbonate	0.74×10^{-2}	-0.17×10^{-2}	1.7×10^{-2}	**
Manganese	-3.0×10^{-2}	-5.3×10^{-2}	-0.72×10^{-2}	**
**Spatial: theta = 1.61 ± 0.39**				
(Intercept)	11.5	11.2	11.8	
MEM2	0.58	0.28	0.88	***
MEM4	-0.40	-0.74	-0.06	**
**Environment + Spatial: theta = 1.76 ± 0.43**				
(Intercept)	10.7	9.9	11.6	
MEM2	0.45	0.15	0.76	**
MEM4	-0.32	-0.66	0.03	*

* $p<0.05$,

** $p<0.01$

*** $p<0.001$

Table S5. CFDA Microbial Cell Count Data: Coefficients and 5% Confidence Intervals from the Negative Binomial GLM for the Correlation with the Explanatory Variables: Environment and Space.

	Coefficient	Confidence Interval		Significance
		2.5%	97.5%	
**Environment: theta = 1.21 ± 0.39**				
(Intercept)	10.0	9.4	10.7	
Dissolved organic carbon	2.6 x 10-2	-5.5 x 10-3	5.8 x 10-2	**
Manganese	-2.7 x 10-2	-4.5 x 10-2	-6.1 x 10-3	*
**Spatial: theta = 1.38 ± 0.33**				
(Intercept)	9.9	9.6	10.2	
MEM2	0.36	0.01	0.72	*
MEM4	-0.32	-0.63	-0.01	*
MEM7	0.47	0.13	0.80	**
**Environment + Spatial: theta = 1.55 ± 0.38**				
(Intercept)	10.0	9.4	10.6	
manganese	-2.73E-02	-4.52E-02	-6.55E-03	**
MEM7	5.12E-01	2.01E-01	8.02E-01	***

* $p<0.05$,

** $p<0.01$

*** $p<0.001$
